# Absence of GIP secretion alleviates age-related obesity and insulin resistance

**DOI:** 10.1530/JOE-19-0477

**Published:** 2020-01-23

**Authors:** Yoshinori Kanemaru, Norio Harada, Satoko Shimazu-Kuwahara, Shunsuke Yamane, Eri Ikeguchi, Yuki Murata, Sakura Kiyobayashi, Tomonobu Hatoko, Nobuya Inagaki

**Affiliations:** 1 Department of Diabetes, Endocrinology and Nutrition, Graduate School of Medicine, Kyoto University, Kyoto, Japan; 2 Preemptive Medicine and Lifestyle Related Disease Research Center, Kyoto University Hospital, Kyoto, Japan

**Keywords:** aging, obesity, incretin, GIP

## Abstract

Glucose-dependent insulinotropic polypeptide (GIP) is an incretin secreted from enteroendocine K cells after nutrient ingestion. Fat strongly induces GIP secretion, and GIP hypersecretion is involved in high-fat diet-induced obesity and insulin resistance. Aging also induces GIP hypersecretion, but its effect on body weight gain and insulin sensitivity remains unclear. In the present study, we investigated the effect of GIP on age-related body weight gain and insulin resistance using GIP-knockout homozygous (GIP^−/^^−^) and heterozygous (GIP^+/^^−^) mice, which have entirely absent and 50% reduced GIP secretion compared to wild-type (WT) mice, respectively. Under 12% fat-containing normal diet feeding condition, body weight was significantly lower in GIP^−/^^−^ mice compared to that in WT and GIP^+/^^−^ mice from 38 weeks of age, while there was no significant difference between WT and GIP^+/^^−^ mice. Visceral and s.c. fat mass were also significantly lower in GIP^−/^^−^ mice compared to those in WT and GIP^+/^^−^ mice. During oral glucose tolerance test, blood glucose levels did not differ among the three groups. Insulin levels were significantly lower in GIP^−/^^−^ mice than those in WT and GIP^+/^^−^ mice. During insulin tolerance test, GIP^−/^^− ^mice showed higher insulin sensitivity than that of WT and GIP^+/^^−^ mice. Adiponectin mRNA levels were increased and leptin mRNA levels tended to be decreased in adipose tissue of GIP^−/^^−^ mice. These results demonstrate that GIP is involved in age-related obesity and insulin resistance and that inhibition of GIP secretion alleviates age-related fat mass gain and insulin resistance under carbohydrate-based diet feeding condition.

## Introduction

Life expectancy has increased in developed countries and is accompanied by the main age-related changes in body composition, which are an increase in fat mass and a decrease in muscle mass. In addition, visceral fat accumulation causes insulin resistance through inflammation (Kalyani *et al*. 2014). This age-related change is called ‘sarcopenic obesity’, and is an important health issue in aging societies (Prado *et al*. 2012, Cleasy *et al*. 2016). Obesity is related to a decline of activities of daily living (ADL) in elderly people and is also related to the high prevalence of medical disorders such as diabetes, hyperlipidemia, and hypertension (Cheng *et al*. 2013, Chang *et al*. 2017). It is therefore important for elderly people to prevent excessive fat accumulation with aging.

Glucose-dependent insulintropic polypeptide/gastric inhibitory polypeptide (GIP) is an incretin secreted from enteroendocrine K cells in response to glucose and fat ingestion and enhances glucose-dependent insulin secretion through the GIP receptor (GIPR) expressed in pancreatic β-cells (Seino *et al*. 2013). GIPR is expressed in adipose tissue as well (Joo *et al*. 2017). GIP plays an important role in high-fat diet (HFD)-induced obesity and insulin resistance (Harada *et al*. 2008, Joo *et al*. 2017). Previous studies using GIP immunoneutralization, GIPR-knockout mice, and GIPR antagonists reported that inhibition of GIP signaling ameliorates HFD-induced obesity and insulin resistance (Miyawaki *et al*. 2002, McClean *et al*. 2008, Boylan *et al*. 2015). HFD strongly stimulates GIP secretion (Iwasaki *et al*. 2015, Sankoda *et al*. 2017, Murata *et al*. 2019), and inhibition of GIP secretion also alleviates HFD-induced obesity and insulin resistance (Nasteska *et al*. 2014). Previous human study found that GIP secretion after glucose ingestion is increased in elderly subjects (Garduno-Garcia *et al*. 2018). We previously reported that aged mice exhibit not only GIP hypersecretion but also excessive fat mass and insulin resistance under normal diet feeding condition (Ikeguchi *et al*. 2018). These results indicate that GIP hypersecretion from K cells may be involved in age-related fat mass gain and insulin resistance. However, the effect of age-related GIP hypersecretion on body weight and fat mass gain, and insulin resistance remains unclear. In this study, we investigated the effect of entirely absent and 50% reduced GIP secretion on age-related body weight, body fat composition, glucose tolerance, and insulin sensitivity under carbohydrate-based normal diet feeding condition using GIP-knockout mice.

## Materials and methods

### Animals

GIP-knockout mice were generated previously (Nasteska *et al*. 2014). GIP secretion was entirely absent in homozygous (GIP^−/^^−^) mice and was reduced in heterozygous (GIP^+/^^−^) mice by 50% compared with that in wild-type (WT) mice, respectively. Male GIP^−/^^−^, GIP^+/^^−^ and WT littermate mice were used in all experiments. Aged mice were defined as age 1 year (50–60 weeks) as described previously (Ikeguchi *et al*. 2018). Experiments were carried out in three separate cohorts, each consisting of three groups of five to seven mice. Body weight and fat weight were evaluated and oral glucose tolerance test (OGTT) was performed in the first cohort. Blood samples under non-fasting condition were collected, and body fat composition analyzed by CT scan, locomotor activity, insulin sensitivity determined by insulin tolerance test (ITT), and measurement of GIP and GLP-1 content in intestine were evaluated in the second cohort. Energy expenditure, food intake, and gene expression were evaluated in the third cohort. The mice were housed in an air-controlled 25°C room with a dark-light cycle of 10 and 14 h with free access to water and normal diet food (3.73 kcal/g; 12% fat, 23% protein, and 65% carbohydrate; Funabashi Farm, Funabashi, Japan). Animal care and procedures were approved by Kyoto University Animal Care Committee (MedKyo15298).

### Blood samples

50 µL blood samples were collected from the tail vein at 10:00 h under non-fasting condition. After a 16-h fasting period, OGTTs (2 g glucose/body weight (kg) for blood glucose levels, plasma insulin and total GIP levels, and 6 g glucose/body weight (kg) for plasma glucagon-like peptide-1 (GLP-1) levels) were performed. Blood samples were collected from the tail vein at 0, 15, 30, 60, and 120 min after oral glucose administration by oral gavage. Blood glucose levels were measured by the glucose oxidase method (Sanwa Kagaku Kenkyusho, Nagoya, Japan). Plasma insulin, total GIP levels, and GLP-1 levels were measured by insulin ELISA kit (Shibayagi, Gunma, Japan), total GIP ELISA kit (Millipore Corporation), and V-PLEX GLP-1 Total Kit (MESO SCALE DISCOVERY, Rockville, MD, USA), respectively. For ITT, human regular insulin (Eli Lilly and Company) at a dose of 0.75 U insulin/body weight (kg) was injected to the intraperitoneal cavity after a 4-h fasting period. Blood glucose levels were measured at 0, 30, 60, 90, and 120 min after insulin administration.

### GIP and GLP-1 content in intestine

Small intestine and colon (4 cm length) were taken from the mice. Samples were extracted with 1 mL of 0.2 N perchloric acid and were centrifuged for 15 min at 20,000 ***g*** at 4°C. The supernatant was used for measurement of GIP and GLP-1 content in intestine as previously described (Ikeguchi *et al*. 2018).

### Measurement of body fat composition

After the mice were dissected, the weights of visceral fat (both sides of epididymal fat), s.c. fat (both sides of inguinal s.c. fat), and the right side of the gastrocnemius muscle were measured. Body fat mass, lean body mass, and fat content in liver were measured using a La Theta experimental animal CT scan system (LCT-100M, Hitachi Aloka Medical). Contiguous 2 mm slice images from the top of the diaphragm to the caudal region were used for quantitative analysis of fat mass and lean body mass (visceral mass without visceral fat mass and s.c. fat mass) of each mouse by La Theta software (vs. 3.00). Fat content in the liver was calculated from density data of fat (100% fat) and muscle (0% fat).

### Energy expenditure and locomotor activity

Energy expenditure was calculated using an Alco System model 2000 (Alco System, Chiba, Japan). Locomotor activity was monitored as distance in a standardized locomotor chamber box (15 × 35 × 40 cm). After the mice were placed into the tracking box for 48 h, locomotor activity was monitored for 24 h using SMART Video Tracking System (Panlab SL, Barcelona, Spain) with free access to water and diet.

### Quantitative RT-PCR

Total RNAs of the islets, small intestine, s.c. fat, and visceral fat were extracted using RNeasy Mini Kit (Qiagen) and TRIzol Reagant (Invitrogen). For cDNA synthesis, RNA was reverse-transcribed using a PrimeScript RT reagent kit (Takara Bio). The mRNA expression levels were measured by quantitative RT-PCR using the ABI PRISM 7000 Sequence Detection System (Applied Biosystems). SYBR Green PCR Master Mix (Applied Biosystems) was prepared for the PCR run. β-Actin was used as the internal control. Each data point of mRNA expression was standardized against β-actin. Primer pairs for PCR were designed previously (Joo *et al*. 2017, Ikeguchi *et al*. 2018).

### Statistical analysis

All data are expressed as the mean ± s.e. Statistical analysis was carried out using one-way ANOVA with the Tukey–Kramer multiple comparison tests. *P* values <0.05 were considered significant.

## Results

### Effect of absent and reduced GIP secretion on body weight gain with aging

**Figure 1:**
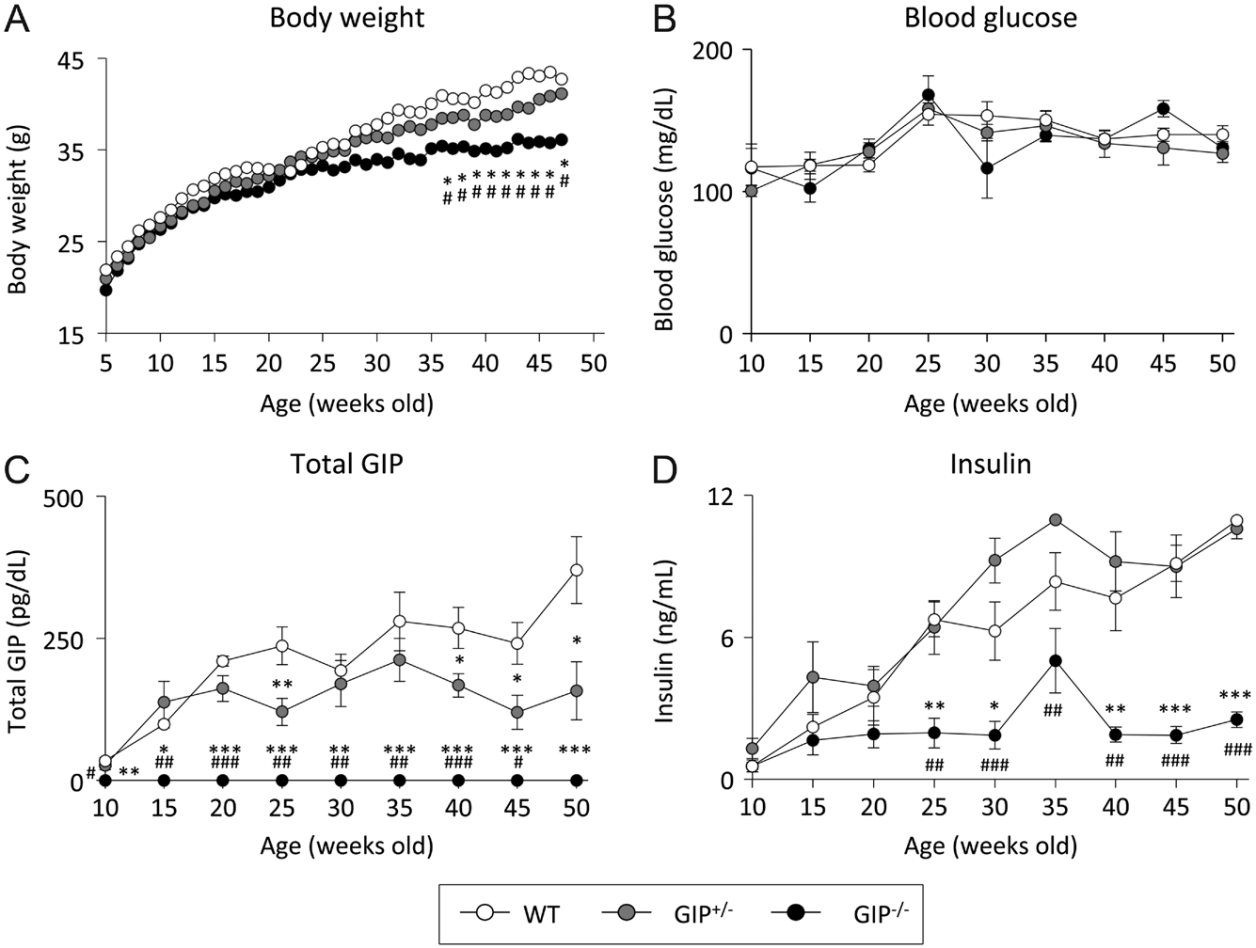
Body weight gain and non-fasting blood glucose levels, GIP levels, and insulin levels in GIP^–/^^–^ and GIP^+/^^–^ mice. (A) Body weight, (B) blood glucose levels, (C) total GIP levels, and (D) insulin levels (*n* = 6–7). WT mice (white circles), GIP^+/^^−^ mice (gray circles), and GIP^−/^^−^ mice (black circles). ***P < 0.001 vs WT mice. ^#^*P* < 0.05, ^##^*P* < 0.01, ^###^*P* < 0.001 vs GIP^+/^^−^ mice.

Body weight was significantly decreased in GIP^–/^^–^ mice compared to that in WT and GIP^+/^^–^ mice from 38 weeks of age, but there was no difference between WT and GIP^+/^^–^ mice (Fig. 1A). Non-fasting glucose, total GIP, and insulin levels were measured once each 5 weeks. There was no difference in blood glucose levels (Fig. 1B) among the three groups. Total GIP levels were increased with aging in both WT and GIP^+/^^–^ mice (Fig. 1C). Total GIP levels were lower in GIP^+/^^–^ mice compared to those in WT mice. Total GIP levels were not detectable in GIP^–/^^–^ mice. Insulin levels were increased with aging in WT and GIP^+/^^–^ mice (Fig. 1D), but insulin levels were significantly lower in GIP^–/^^–^ mice compared to those in WT and GIP^+/^^–^ mice from 25 weeks of age.

### Effect of absent and reduced GIP secretion on body fat mass

**Figure 2:**
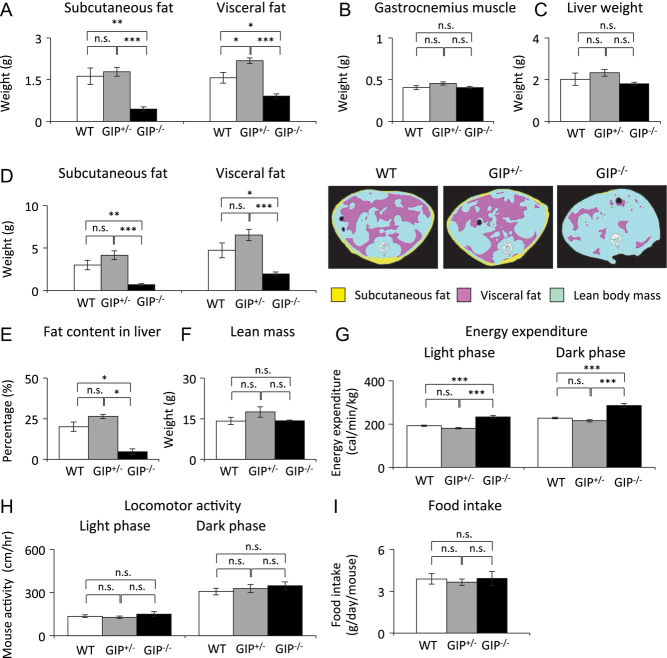
Body composition, energy expenditure, locomotor activity, and food intake in GIP^−/^^−^ and GIP^+/^^−^ mice. (A) Subcutaneous and visceral fat weight, (B) gastrocnemius muscle weight, and (C) liver weight in 60-week-old mice (*n* = 5−6). (D) Subcutaneous and visceral fat mass, (E) lean mass, and (F) fat content in liver estimated by CT scan in 58-week-old mice (*n* = 5–6). Pink, yellow, and blue areas represent visceral fat, subcutaneous fat, and lean mass, respectively. (G) Energy expenditure in 51-week-old mice (*n* = 6). (H) Locomotor activity in 54-week-old mice (*n* = 6). (I) Food intake in 52-week-old mice (*n* = 6). WT mice (white bars), GIP^+/^^−^ mice (gray bars), and GIP^−/^^−^ mice (black bars). *P < 0.05, **P < 0.01, ***P < 0.001. n.s., no significant difference.

Subcutaneous and visceral fat weights were significantly lower in GIP^−/^^−^ mice compared to those in WT and GIP^+/^^−^ mice (Fig. 2A). There was no significant difference in fat weight between WT and GIP^+/^^−^ mice. Gastrocnemius muscle and liver weight did not differ among the three groups (Fig. 2B and C). CT scan analysis showed that GIP^−/^^−^ mice had significantly lower body fat mass and fat content in liver compared to those in WT and GIP^+/^^−^ mice (Fig. 2D and E), but there was no significant difference between WT and GIP^+/^^−^ mice. Lean body mass did not differ among the three groups (Fig. 2F). Energy expenditure was significantly higher in GIP^−/^^−^ mice compared to that in WT and GIP^+/^^−^ mice (Fig. 2G), but did not differ between WT and GIP^+/^^−^ mice. Locomotor activity and food intake did not differ among the three groups (Fig. 2H and I).

### Effect of absent and reduced GIP secretion on glucose tolerance and insulin sensitivity

**Figure 3:**
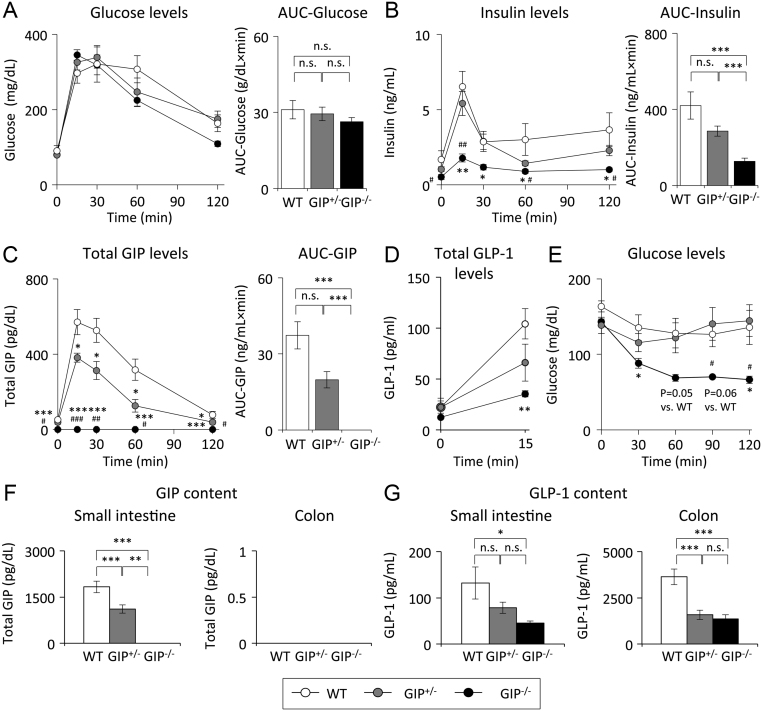
Oral glucose tolerance test (OGTT) and insulin tolerance test (ITT). (A) Blood glucose levels, (B) insulin levels, and (C) total GIP levels during OGTT (2 g glucose/kg body weight) in 52-week-old mice (*n* = 6). (D) Total GLP-1 levels during OGTT (6 g glucose/kg body weight) in 54 weeks old mice (*n* = 6). (E) Blood glucose levels during ITT (0.75 U insulin/kg body weight) in 54 weeks old mice. (F) GIP content in small intestine and colon in GIP^−/^^−^ and GIP^+/^^−^ in 54 weeks old mice (*n* = 6). (G) GLP-1 content in small intestine and colon in GIP^−/^^−^ and GIP^+/^^−^ in 54 weeks old mice (*n* = 6). WT mice (white bars and circles), GIP^+/^^−^ mice (gray bars and circles), and GIP^−/^^−^ mice (black bars and circles). *P < 0.05, **P < 0.01, ***P < 0.001 vs WT mice. ^#^*P* < 0.05, ^##^*P* < 0.01, ^###^*P* < 0.001 vs GIP^+/^^−^ mice. n.s., no significant difference. AUC, area under the curve.

During OGTT, blood glucose levels were not significantly different among the three groups (Fig. 3A). Insulin levels were remarkably lower in GIP^−/^^−^ mice compared to those in WT and GIP^+/^^−^ mice (Fig. 3B). Insulin levels did not differ between WT and GIP^+/^^−^ mice. Total GIP levels were reduced and not detectable in GIP^+/^^−^ and GIP^−/^^−^ mice, respectively (Fig. 3C). GIP secretion (area under the curve of GIP during OGTT) in GIP^+/^^−^ mice was reduced by 50% compared to that in WT mice. Total GLP-1 levels at 15 min were significantly lower in GIP^−/^^−^ mice than those in WT mice (Fig. 3D); total GLP-1 levels did not differ between WT and GIP^+/^^−^ mice. During ITT, blood glucose levels were lower in GIP^−/^^−^ mice compared with those in WT and GIP^+/^^−^ mice (Fig. 3E). Insulin sensitivity did not differ between WT and GIP^+/^^−^ mice. GIP content in small intestine was reduced and not detectable in GIP^+/^^−^ and GIP^−/^^−^ mice, respectively (Fig. 3F). GIP content in small intestine was significantly smaller in GIP^−/^^−^ mice than those in WT and GIP^+/^^−^ mice. GIP content was not detected in colon in any of the three groups of mice. GLP-1 content was smaller in the small intestine and colon of GIP^−/^^−^ mice than that in WT and GIP^+/^^−^ mice (Fig. 3G).

### Effect of absent and reduced GIP secretion on GIPR mRNA expression

**Figure 4:**
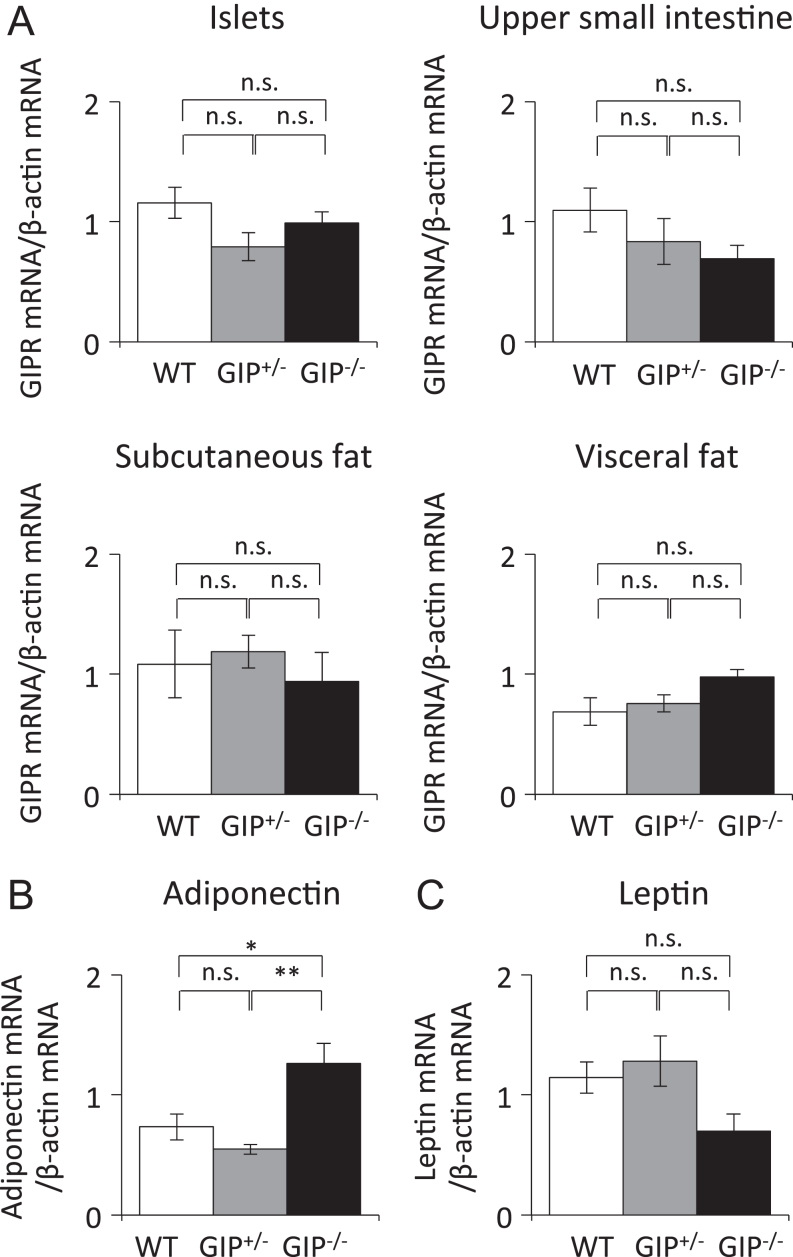
Gene expression in islets, upper small intestine, and adipose tissue in GIP^−/^^−^ and GIP^+/^^−^ mice. (A) Expression levels of GIPR mRNA in islets, upper small intestine, subcutaneous fat, and visceral fat in 54 weeks old mice (*n* = 6). Expression levels of (B) adiponectin mRNA and (C) leptin mRNA expression in visceral fat in 54 weeks old mice (*n* = 6). WT mice (white bars), GIP^+/^^−^ mice (gray bars), and GIP^−/^^−^ mice (black bars). *P < 0.05 and **P < 0.01. n.s., no significant difference.

GIPR was reported to be expressed in islets, small intestine, and adipose tissue (Usdin *et al*. 1993, Seino *et al*. 2013, Joo *et al*. 2017), and the expression levels of GIPR mRNA did not differ among the three groups (Fig. 4A). Expression levels of adiponectin mRNA in adipose tissue were significantly higher in GIP^−/^^−^ mice compared with those in WT and GIP^+/^^−^ mice (Fig. 4B). Expression levels of leptin mRNA tended to be decreased in GIP^−/^^−^ mice, but there was no significant difference among the three groups (Fig. 4C).

## Discussion

Both insulin and total GIP levels are increased under HFD-induced obese condition (Nasteska *et al*. 2014, Shimazu-Kuwahara *et al*. 2017, Murata *et al*. 2019). We previously investigated the effects of absent and 50% reduced GIP secretion on HFD-induced obesity and insulin resistance using GIP-knockout mice; we found that reduced GIP secretion as well as absent GIP secretion alleviates fat mass gain and insulin resistance under HFD feeding condition (Nasteska *et al*. 2014). These results demonstrate that reduced GIP secretion as well as absent GIP secretion contribute to alleviation of HFD-induced obesity and insulin resistance. Insulin and total GIP levels are increased under aged condition (Trahair *et al*. 2012, Garduno-Garcia *et al*. 2018, Ikeguchi *et al*. 2018). In the present study, the effects of absent and reduced GIP secretion on age-related fat mass gain and insulin resistance under normal fat diet feeding condition were investigated using the same GIP-knockout mouse used in the previous study. We found that GIP secretion is increased with aging in WT and GIP^+/^^−^ mice and that absence of GIP secretion as shown in GIP^−/^^−^ mice alleviates age-related body weight and fat mass gain and insulin resistance, indicating that GIP hypersecretion is associated with age-related body weight gain and insulin resistance under normal fat diet feeding condition. On the other hand, reduction of GIP secretion as shown in GIP^+/^^−^ mice did not affect body weight and fat mass gain, and insulin resistance under normal fat diet feeding condition. Previous study using GIPR-knockout mice also showed that the effect of GIP signaling on body weight gain and insulin resistance is less under high-carbohydrate diet feeding condition than it is under HFD feeding condition (Maekawa *et al*. 2018). The study suggests that these effects of GIP reflect the different effects of carbohydrate diet and HFD on body weight and insulin sensitivity. HFD induces more body weight gain and fat mass gain than carbohydrate diet, primarily because fat has a higher caloric value than the same amount of carbohydrate (Jois *et al*. 2016). HFD is also associated with unfavorable changes in the type and number of gut bacteria and bile acids composition in the intestine, which induce obesity and insulin resistance (Hildebrandt *et al*. 2009, Islam *et al*. 2011, Yokota *et al*. 2012, Saad *et al*. 2016). In addition, GIP potentiates insulin secretion from pancreatic β-cells as an incretin and thus plays an important role in hyperinsulinemia under HFD-feeding obese condition (Harada *et al*. 2008). GIP also increases IL-6 expression and production in adipocytes in the presence of TNF-α, which is induced by obesity and enhances HFD-induced insulin resistance through IL-6 signaling (Joo *et al*. 2017). These findings demonstrate that the obesity and insulin resistance under HFD feeding condition are accelerated by GIP signaling compared to those under carbohydrate-based normal diet feeding condition. Thus, we might well not be able to find a major difference in body weight and insulin sensitivity between WT and GIP^+/^^−^ mice under normal fat diet feeding condition used in this study.

It was previously reported that aged GIPR-knockout mice show improvement of insulin resistance and reduced fat mass without a reduction of body weight compared to aged WT mice under normal diet feeding condition (Yamada *et al*. 2007). Locomotor activity and lean body mass were increased and body temperature and heart rate were decreased in these aged GIPR-knockout mice. However, in the present study, locomotor activity, lean body mass, and rectal temperature (data not shown) did not differ in WT and GIP^−/^^−^ mice. There are several possible explanations for the difference between the results from the GIPR-knockout mice and our GIP^−/−^ mice. First, the methods of measurement of locomotor activity and body temperature in our study are quite different from those in the previous study. Second, the measurement area used to evaluate fat and lean body mass by CT scan differs between the previous study and our study. Third, housing conditions and mouse microbiota might contribute to the differences in body weight gain. Therefore, a direct comparison study is needed to clarify the difference of phenotype between aged GIPR-knockout and GIP^−/^^−^ mice.

Muscle and liver have much higher contribution to glucose uptake and energy expenditure than adipose tissue (DeFronzo *et al*. 1987, Gallagher *et al*. 1998). Lean body mass should be considered in the study of glucose uptake and energy expenditure. In this study, however, lean body mass was measured by CT scan from the top of the diaphragm to the femoral head. This area mass reflects the weight of internal organs including iliopsoas muscles but not the muscle weight of whole body. Therefore, we performed OGTT in proportion to whole body and calculated energy expenditure by the whole body weight.

Recently, antagonism of the GIP receptor has come to be seen as potential therapeutic target for obesity and insulin resistance (McClean *et al*. 2007, 2008, Boylan *et al*. 2015, Kaneko *et al*. 2019). In this study, GIP is shown to be involved in age-related obesity and insulin resistance under normal fat diet feeding condition. Furthermore, inhibition of GIP secretion might enable us to prevent fat accumulation with aging and declination of ADL levels in the elderly. Interestingly, inhibition of GIP secretion might also have a favorable effect on extension of lifespan; a recent study using GIPR-knockout mice reported that inhibition of GIP signaling is involved in extension of lifespan (Hoizumi *et al*. 2019).

In conclusion, GIP is involved in age-related obesity and insulin resistance, and inhibition of GIP secretion alleviates age-related body weight and fat mass gain, and insulin resistance under carbohydrate-based feeding condition.

## Declaration of interest

The authors declare that there is no conflict of interest that could be perceived as prejudicing the impartiality of the research reported.

## Funding

This study was supported by grants from the Ministry of Education, Culture, Sports, Science and Technology (MEXT), Japan Society for the Promotion of Science (JSPS) (Grant No. 19K09022), Ministry of Health, Labour, and Welfare, Ministry of Agriculture, Forestry and Fisheries, Japan Diabetes Foundation, Japan Association for Diabetes Education and Care, Merck Sharp & Dohme (MSD) Life Science Foundation, Public Interest Incorporated Foundation, Japan Diabetes Foundation, and Suzuken Memorial Foundation.

## Author contribution statement

Y K and N H planned the study, researched data, contributed to discussion, wrote, reviewed and edited the manuscript. S S-K planned the study and researched data. S Y, E I, Y M, S K, and T H researched data. N I planned the study, contributed to discussion, and edited the manuscript. All authors approved the final version of the manuscript.
